# Production of Deglucose-Apiose-Xylosylated Platycosides from Glycosylated Platycosides by Crude Enzyme from *Aspergillus tubingensis*

**DOI:** 10.4014/jmb.2112.12020

**Published:** 2022-02-17

**Authors:** Kyung-Chul Shin, Tae-Geun Kil, Su-Hwan Kang, Deok-Kun Oh

**Affiliations:** 1 Department of Integrative Bioscience and Biotechnology, Konkuk University, Seoul 05029, Republic of Korea; 2 Department of Bioscience and Biotechnology, Konkuk University, Seoul 05029, Republic of Korea

**Keywords:** Platycoside, *Aspergillus tubingensis*, crude enzyme, biotransformation, deglucose-apiose-xylosylated platycoside

## Abstract

Platycosides, Platycodi radix (*Platycodon grandiflorus* root) saponins, are used as food supplements and exert diverse pharmacological activities. Deglycosylation of saponins enhances their biological efficacy, and deglycosylated platycosides are produced mainly through enzymatic hydrolysis. However, the types of available deglycosylated platycosides remain limited because of a lack of hydrolyzing enzymes that can act on specific glycosides in glycosylated platycosides. In this study, a crude enzyme from *Aspergillus tubingensis* converted platycoside E (PE) and polygalacin D_3_ (PGD3) into deglucose-apiose-xylosylated (deGAX)-platycodin D (PD) and deGAX-polygalacin D (PGD), respectively. The products were identified through LC/MS analysis by specifically hydrolyzing all glucose residues at C-3, and apiose and xylose residues at C-28 of platycoside. The hydrolytic activity of the crude enzyme obtained after the cultivation of the fungus using citrus pectin and corn steep solid as carbon and nitrogen sources, respectively, in culture medium was increased compared with those using other carbon and nitrogen sources. The crude enzyme from *A. tubingensis* was the most effective in producing deGAX platycoside at pH 5.0 and 60°C. The crude enzyme produced 0.32 mg/ml deGAX-PD and 0.34 mg/ml deGAX-PGD from 1 mg/ml PE and 1 mg/ml PGD3 (at pH 5.0 and 60°C) for 12 and 10 h, with productivities of 32.0 and 42.5 mg/l/h and molar yields of 62.1 and 59.6%, respectively. To the best of our knowledge, this is the first study to produce deGAX platycosides from glycosylated platycosides.

## Introduction

Platycoside is a pharmacologically active saponin present in Platycodi radix, the root of *Platycodon grandiflorus*, known as bell flower, or balloon flower [1]. Over the past decade, interest in platycosides has increased because of their diverse nutraceutical and pharmacological properties which include anti-inflammatory [2, 3], antioxidant [4, 5], immunoregulatory [6-8], anti-tumor [9-11], and anti-obesity effects [12-14]. Platycosides are triterpenoids composed of two side chains with sugar moieties, including the glucose (glucopyranosyl-glucopyranosyl-glucopyranosyl) moiety at C-3 and the oligosaccharide (apiofuranosyl-xylopyranosyl-rhamnopranosyl-arabinopyranosyl) moiety at C-28. Most platycosides in Platycodi radix belong to platycodigenin-type platycosides, including platycodin E (PE), platycodin D_3_ (PD3), platycodin D (PD), deapiosylated (deA)-PE, deA-PD3, and deA-PD, and polygalacic acid-type platycosides, including polygalacin D_3_ (PGD3) and polygalacin D (PGD), which account for approximately 50% and 30% of all platycosides, respectively [15, 16].

Saponins are deglycosylated to exert stronger biological effects by enhancing their bioavailability and cell permeability [17, 18] using different methods such as heating [19], acid treatment [20], and enzymatic hydrolysis [21]. As enzymatic hydrolysis provides higher specificity, efficiency, and yield than any other method, it is the one most often employed for the deglycosylation of platycosides. The glucose moiety at C-3 of platycoside is hydrolyzed by β-glucosidases from *Aspergillus niger* as the commercial enzyme Pluszyme 2000P [22], *Aspergillus usamii* [23], *Caldicellulosiruptor owensensis* [24], *Caldicellulosiruptor bescii* [25], and *Dictyoglomus turgidum* [26], laminarinase from *Trichoderma* sp. [27], snailase (a complex of cellulase, hemicellulase, pectinase, and β-glucuronidase) from snails [28], and cellulase from *Trichoderma reesei* [29], which showed no activity for the oligosaccharide moiety at C-28. In contrast, the crude enzymes from *A. niger* [30] and *Rhizopus oryzae* [31], and pectinase from *A. niger* as the commercial enzyme Cytolase PCL5 [32] hydrolyze not only the glucose moiety at C-3 but also the oligosaccharide moiety at C-28; however, none of these enzymes hydrolyze the innermost glucopyranosyl residue at C-3.

**Fig. 1:**
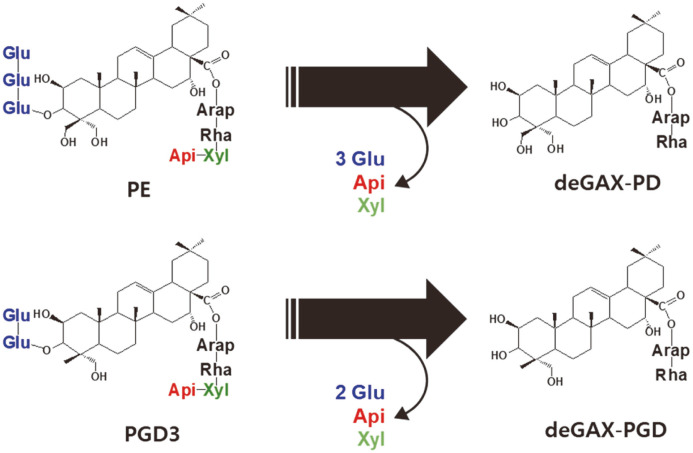
Biotransformation of PE and PGD3 into deGAX-PD and deGAX-PGD, respectively, by the crude enzyme from *A. tubingensis*. Arap, α-L-arabinopyranosyl-; Rham, α-L-rhamnopyranosyl-; Xyl, β-D-xylopyranosyl- (green); Api, β-D-apiofuranosyl- (red); and Glu, β-D-glucopyranosyl- (blue).

In this study, we found that a crude enzyme from *Aspergillus tubingensis*, a generally recognized as safe (GRAS) fungus, hydrolyzed all glucose residues at C-3, and apiose and xylose residues at C-28 of platycoside to produce deglucose-apiose-xylosylated (deGAX) platycosides (Fig. 1). The chemical structures of deGAX platycosides were also identified in this study. For inducing the efficient glycoside-hydrolyzing crude enzyme, citrus pectin and corn steep solid were used as carbon and nitrogen sources, respectively, for the cultivation of the fungus. We used the induced enzyme for the production of deGAX-PD and deGAX-PGD from PE and PGD3, which are the major components of Platycodi radix, respectively.

## Materials and Methods

### Materials

*A. tubingensis* KCTC 14166 (Korean Collection for Type Cultures, Republic of Korea) was used to hydrolyze glycosylated saponins [33]. Platycoside standards, including PE, PD3, PD, deA-PD, PGD3, and PGD, were purchased from Ambo Laboratories (Republic of Korea). Other platycoside standards, such as deglucosylated (deG)-PD and deG-PGD; deapiose-xylosylated (deAX)-PD and deAX-PGD; deGAX-PD and deGAX-PGD; and deA-PGD, deglucose-apiosylated (deGA)-PD, and deGA-PGD, were prepared by purifying the products obtained from the conversions of PD and PGD using Pluszyme 2000P [22], Cytolase PCL5 [32], and the crude enzyme from *A. tubingensis* and those of PGD, deG-PD, and deG-PGD using the crude enzyme from *R. oryzae* [31], respectively. The prepared platycosides were purified using the preparative high-performance liquid chromatography (Prep-HPLC) (Agilent 1260, USA) with a Hydrosphere C18 preparative column (10 × 250 mm, 5 μm particle size; YMC, Japan). The column was eluted with water at a flow rate of 4.7 ml/min at 30°C, and the eluent was monitored using a UV detector at an absorbance of 203 nm. The purified platycosides were used as the standards.

### Culture Conditions

*A. tubingensis* was incubated in a plate containing potato dextrose agar (PDA, Difco, USA) at 25°C for 7 days. After incubation, the spore suspension was harvested by adding sterile distilled water containing 0.85% NaCl and 0.1% (v/v) Triton X-100 to the plate. The spore suspension was subsequently filtered using a sterilized gauze, and the number of spores in the filtered suspension was counted using a hemocytometer (INCYTO, Republic of Korea). The fungal spores were inoculated into 5 ml potato dextrose broth (PDB) in a glass tube at a final concentration of 1.0 × 106 spores/ml. The suspension was cultivated at 25°C with agitation rate of 150 rpm in a shaking incubator for 1 day. After cultivation, mycelia were collected by centrifugation at 13,000 ×*g* at 4°C for 10 min by removing the supernatant. The collected mycelia were washed with 0.85% (w/v) NaCl solution to remove the PDB. The washed mycelia were transferred into 100 ml culture medium in a 500 ml baffled Erlenmeyer flask, and then the culture was incubated at 26°C for 6 days with the agitation rate of 150 rpm in a shaking incubator.

### Culture Medium and Induction

The culture medium contained 20 g/l carbon source, 3–18 g/l nitrogen source, 2 g/l KH_2_PO_4_, 0.3 g/l MgSO_4_·7H_2_O, 0.3 g/l CaCl_2_, 5 mg/l FeSO_4_·7H_2_O, 1.4 mg/l ZnSO_4_·7H_2_O, 1.3 mg/l MnSO_4_·H_2_O, and 3.7 mg/l CoCl_2_·6H_2_O. To examine the effects of carbon and nitrogen sources on the induction of deGAX platycoside-producing activity of the crude enzyme from *A. tubingensis*, 20 g/l arabic gum, cellulose, citrus pectin, rice bran, sugar beet sludge, or wheat bran, and 4 g/l ammonium nitrate, 4 g/l ammonium sulfate, 10 g/l corn steep solid, 9 g/l peptone, 3 g/l urea, or 18 g/l yeast extract with the same concentration of nitrogen were used as carbon and nitrogen sources, respectively. The optimal concentrations of citrus pectin and corn steep solid used for induction were determined by varying the concentration from 5 to 20 g/l and 1 to 9 g/l, respectively. The crude enzyme was obtained after cultivation for 6 days, and the reactions were conducted at 60°C for 1 h in 0.2 M citrate-phosphate buffer (pH 5.0) containing 0.2 mg/ml crude enzyme and 0.4 mg/ml PE.

### Enzyme Preparation

The culture broth (100 ml) was filtered through a Whatman filter paper No. 2, and a 3-fold volume of methanol was added to the filtrate to precipitate the proteins. Afterward, the precipitate was collected by centrifugation at 13,000 ×*g* for 20 min at 4°C and resuspended in 0.2 M citrate-phosphate buffer (pH 5.5). The suspension was dialyzed using a dialysis tube and then concentrated using a 10 kDa cut-off centricon (Amicon Ultra-15; Millipore, USA). The concentrated solution was used as the crude enzyme after its protein content was quantified using the Bradford assay.

### Production of deGAX Platycosides from Glycosylated Platycosides

The effects of pH and temperature on the deGAX platycoside-producing activity of the crude enzyme from *A. tubingensis* were investigated by varying the pH from 3.5 to 6.0 at 50°C and temperature from 40 to 65°C at pH 5.0 in 0.2 M citrate-phosphate buffer containing 0.2 mg/ml enzyme and 0.4 mg/ml PE for 1 h. The time-course reactions for the production of deGAX-PD and deGAX-PGD from PE and PGD3 by the crude enzyme from *A. tubingensis* were conducted in 50 mM citrate-phosphate buffer (pH 5.0) containing 1 mg/ml platycoside as the substrate and 0.5 mg/ml enzyme at 60°C for 12 and 10 h, respectively. The experiments were performed in triplicate.

### Analysis of Platycosides

To stop the reaction and extract platycosides, *n*-butanol was added to the reaction solution at a ratio of 1:1. The *n*-butanol fraction of the extract was then separated and evaporated until it was completely dry. The dried residue was dissolved in methanol, and the dissolved platycosides were analyzed using an HPLC system (Agilent 1100, USA) equipped with a hydrosphere C18 column (4.6 × 150 mm, 5 μm particle size; YMC) at an absorbance of 203 nm. The column was eluted at 30°C at a flow rate of 1 ml/min with a gradient of acetonitrile ranging from 10 to 40% (v/v) for 30 min, 40 to 90% for 15 min, 90 to 10% for 5 min, and constant at 10% for 10 min. The calibration curve of the peak areas with respect to platycoside standard concentrations (0.2−1.0 mM) was obtained and used to estimate the concentrations of platycosides.

To identify the chemical structures of all products, LC/MS analysis of the platycosides was performed using an ion trap MS (Thermo-Finnigan LCQ Deca XP Plus; Thermo Scientific, USA) at the National Instrumentation Center for Environmental Management, Seoul National University, Republic of Korea. The samples were ionized using electrospray ionization under 275°C capillary temperature, 30 psi nebulizer gas, 5 kV ion source voltage, 46 V capillary voltage in positive mode, 15 V fragmentor voltage in negative mode, 0.02 min average time for changing the polarity, 0.01 min average scan time, and 35% abundant precursor ions at the collision energy.

## Results and Discussion

### Identification of Unknown Products Obtained from the Conversion of PE and PGD3 by the Crude Enzyme from *A. tubingensis*

**Fig. 2:**
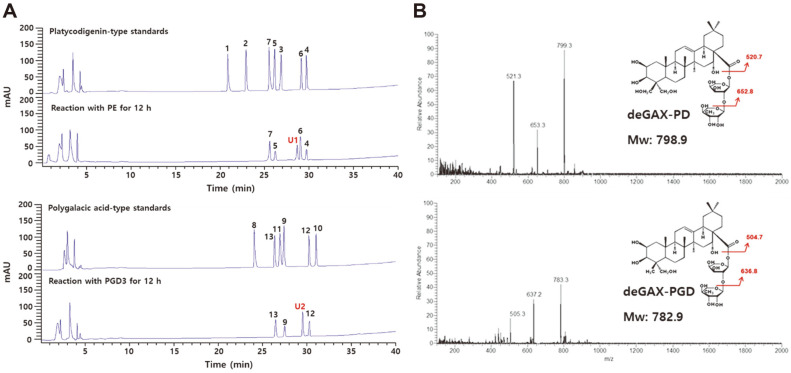
Identification of platycosides obtained from the conversion of PE and PGD3 by the crude enzyme from *A. tubingensis*. (**A**) HPLC chromatograms. (**B**) LC/MS chromatograms. PE (**1**), PD3 (**2**), PD (**3**), deG-PD (**4**), deA-PD (**5**), deGA-PD (**6**), deAX-PD (**7**), PGD3 (**8**), PGD (**9**), deG-PGD (**10**), deA-PGD (**11**), deGA-PGD (**12**), deAX-PGD (**13**), unknown product 1 (**U1**, red), and unknown product 2 (**U2**, red).

The platycosides PE and PGD3 were converted using the crude enzyme from *A. tubingensis*, and the reaction products were identified through HPLC based on the same retention times as that of the platycoside standards, including the platycodigenin-type platycosides PE (**1**), PD3 (**2**), PD (**3**), deG-PD (**4**), deA-PD (**5**), deGA-PD (**6**), and deAX-and PD (**7**); and polygalacic acid-type platycosides PGD3 (**8**), PGD (**9**), deG-PGD (**10**), deA-PGD (**11**), deGA-PGD (**12**), and deAX-PGD (**13**) (Fig. 2A). In the HPLC chromatogram at 12 h reaction time, the products (**4**), (**5**), (**6**), and (**7**); and (**9**), (**12**), and (**13**) obtained from the conversion of PE and PGD3 exhibited the same retention times as deG-PD (29.7 min), deA-PD (26.2 min), deGA-PD (29.1 min), and deAX-PD (25.5 min), and PGD (27.4 min), deGA-PGD (30.2 min), and deAX-PGD (26.3 min), respectively. However, unknown peaks (**U1** and **U2**) were also observed at the retention times of 28.6 and 29.4 min.

To identify the two unknown products, the compounds were analyzed using LC/MS. The total molecular masses of **U1** and **U2** were indicated through distinct peaks at the mass per charge (*m/z*) of 799.3 and 783.3 [M+H]^+^ in the LC/MS spectra (Fig. 2B), which were identical to those of deGAX-PD and deGAX-PGD within *m/z* 0.4, respectively. The molecular masses of the main fragment peaks at *m/z* 653.3 and 520.7 [M+H]^+^ of **U1** or 637.2 and 504.7 [M+H]^+^ of **U2** in the LC/MS spectra were found to be consistent with those obtained from the cleavage of rhamnose and arabinose at C-28 of deGAX-PD or deGAX-PGD, respectively. These results indicated that the crude enzyme from *A. tubingensis* hydrolyzed all three or two glucose residues at C-3 and apiose and xylose at C-28 of PE or PGD3 (Fig. 1), with the products **U1** and **U2** being deGAX-PD and deGAX-PGD, respectively.

### Effects of Carbon and Nitrogen Sources on the Induction of deGAX Platycoside-Producing Activity of the Crude Enzyme from *A. tubingensis*

To induce the deGAX platycoside-producing activity of the crude enzyme from *A. tubingensis*, polysaccharides, including arabic gum, cellulose, citrus pectin, rice bran, sugar beet sludge, and wheat bran, were used as carbon sources (Table 1). Carboxymethyl cellulose (CMC) was used as a control as it was most effective in inducing the hydrolytic activity of *A. niger* for ginseng saponins [34]. The induced deGAX platycoside-producing activity was evaluated by measuring the conversion of PE into deGAX-PD because PE, which had one more glucose molecule than PGD3, was predicted to require higher hydrolytic activity than PGD3. When citrus pectin was added to the medium for the cultivation of the fungus, the deGAX platycoside-producing activity was found to be the highest among the carbon sources evaluated, being 1.33-fold higher than that when CMC was used. In contrast, when cellulose, rice bran, and wheat bran were used, the activities were found to be lower than that when CMC was used.

**Fig. 3:**
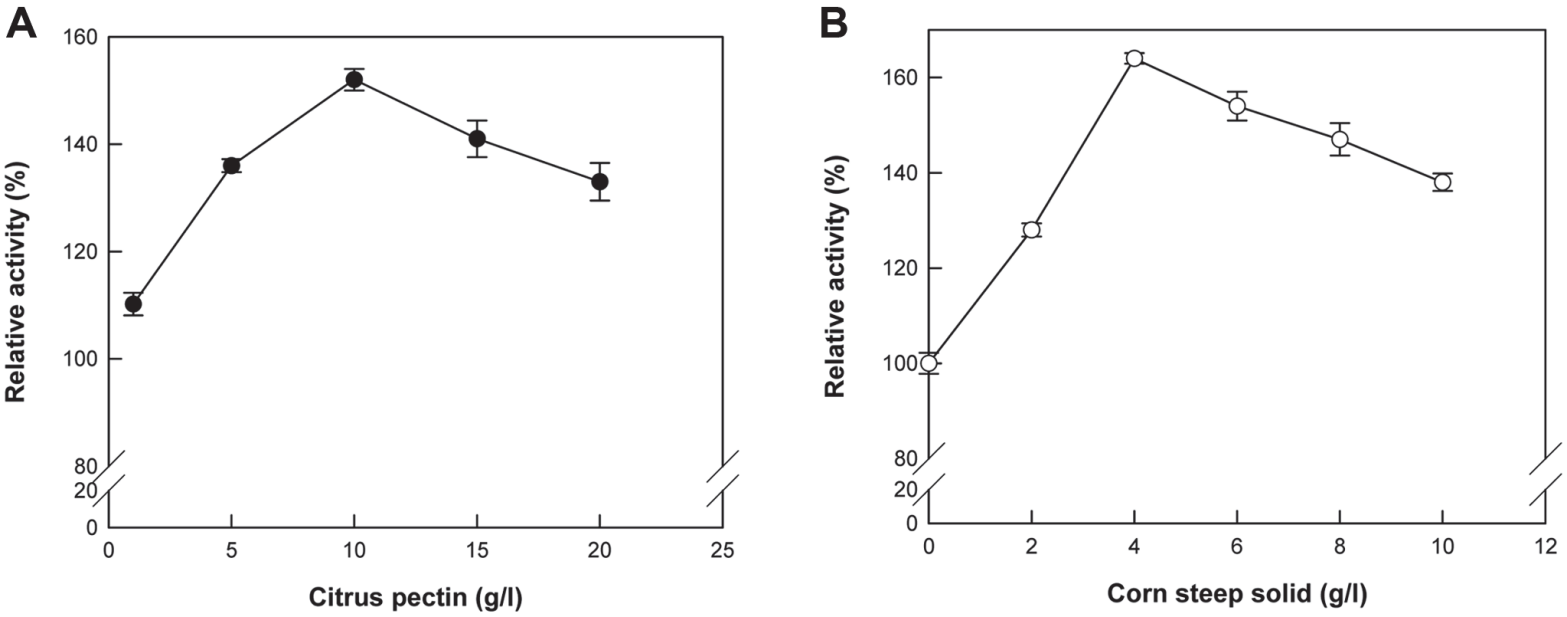
Effects of (A) citrus pectin and (B) corn steep solid concentrations on the deGAX platycosideproducing activity of the crude enzyme from *A. tubingensis*. Data are expressed as the means of three experiments and the error bars represent standard deviations.

Even when arabic gum and sugar beet sludge were used, no activity was observed. Since citrus pectin contains xylose in homogalacturonan and apiose in rhamnogalacturonan-II [35], its induction effect for the deGAX platycoside-producing activity by hydrolyzing glucoside, xyloside, and apioside seems to be better than that of other carbon sources. Thus, the effect of citrus pectin concentration on the induction of deGAX platycoside-producing activity was further investigated, and the maximum activity was observed at 10 g/l citrus pectin (Fig. 3A). Therefore, *A. tubingensis* was cultivated using 10 g/l citrus pectin as a carbon source for inducing the deGAX platycoside-producing activity of the crude enzyme from *A. tubingensis*.

To further increase the deGAX platycoside-producing activity of the crude enzyme from *A. tubingensis*, the effect of the nitrogen source as an inducer was evaluated (Table 1). When corn steep solid was added to the medium to cultivate the fungus, the deGAX platycoside-producing activity was found to be 1.38-fold higher than that obtained without using a nitrogen source. It also showed the highest value among the nitrogen sources evaluated. Peptone also showed a positive effect on induction, whereas ammonium nitrate, ammonium sulfate, urea, and yeast extract exhibited negative effects. The induction effect for the deGAX platycoside-producing activity by complex nitrogen sources such as corn steep solid and peptone may suggest that *A. tubingensis* requires unknown factors for elaborating deGAX platycoside-producing enzymes. Therefore, the deGAX platycoside-producing activity was determined by varying the concentration of corn steep solid. When 4 g/l corn steep solid was used, the activity was found to be maximal, reaching 1.64-fold higher than that obtained without using a nitrogen source (Fig. 3B). Therefore, 4 g/l corn steep solid was used as a nitrogen source for inducing the deGAX platycoside-producing activity.

### Effects of pH and Temperature on the deGAX Platycoside-Producing Activity of the Crude Enzyme from *A. tubingensis*

**Fig. 4:**
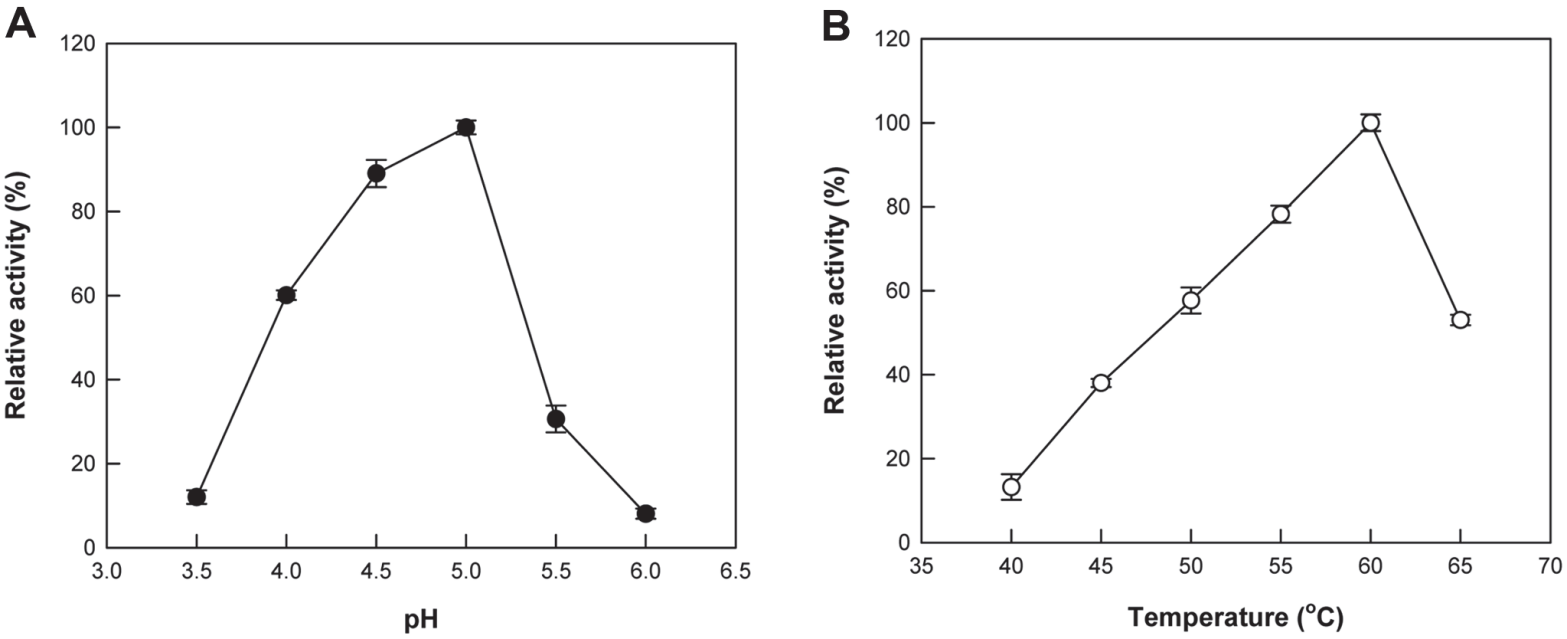
Effects of (A) pH and (B) temperature on the deGAX platycoside-producing activity of the crude enzyme from *A. tubingensis* using PE. Data are expressed as the means of three experiments and the error bars represent standard deviations.

To investigate the effects of pH and temperature on the deGAX platycoside-producing activity of the crude enzyme from *A. tubingensis*, we varied them from 3.5 to 6.0 at 50°C and 40 to 65°C at pH 5.0, respectively, and the conversion of PE into deGAX-PD was evaluated for the same reasons as in the experiments in effects of carbon and nitrogen sources. The maximal activity was observed at pH 5.0 and 60°C. The relative activities were 89 and 30% of the maximal activity at pH 4.5 and 5.5 (Fig. 4A), respectively, and 78 and 53% at 55 and 65°C, respectively (Fig. 4B). The optimal pH and temperatures for the conversion of saponins by the crude enzymes from *A. niger* [34] and *R. oryzae* [31] were pH 5.0 and 55°C; and pH 5.5 and 50°C, respectively.

### Production of deGAX Platycosides from Glycosylated Platycosides by the Crude Enzyme from *A. tubingensis*

**Fig. 5:**
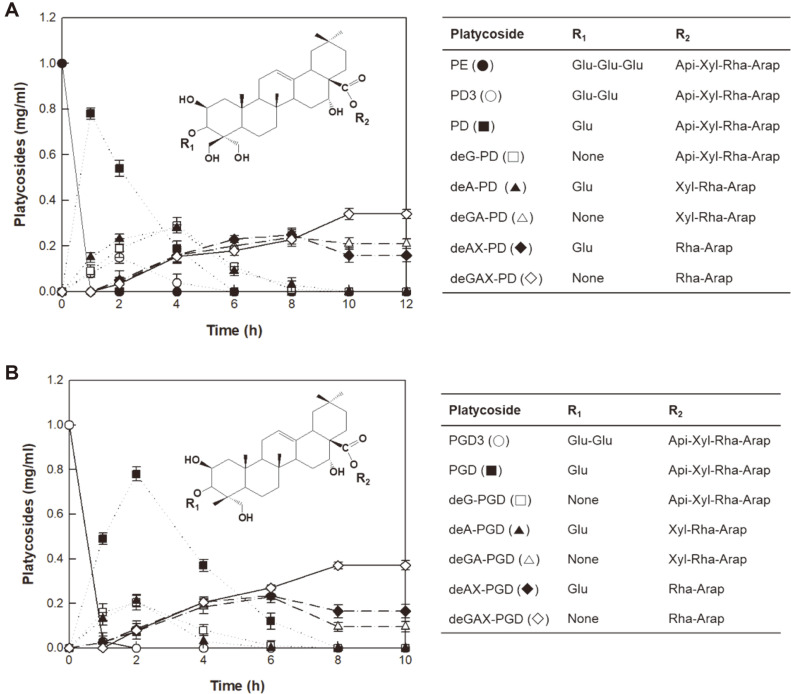
Time-course reactions for the production of deGAX platycosides from glycosylated platycosides by the crude enzyme from *A. tubingensis*. (**A**) Production of deGAX-PD from PE. (**B**) Production of deGAX-PGD from PGD3. Arap, α-L-arabinopyranosyl-; Rham, α-L-rhamnopyranosyl-; Xyl, β-D-xylopyranosyl-; Api, β-D-apiofuranosyl-; and Glu, β-D-glucopyranosyl-. Data are expressed as the means of three experiments and the error bars represent standard deviations.

Time-course reactions for the production of deGAX-PD and deGAX-PGD from reagent-grade PE and PG3, respectively, were performed using the crude enzyme from *A. tubingensis* at pH 5.0 and 60°C. The enzyme produced 0.32 mg/ml (0.401 mM) deGAX-PD from 1 mg/ml (0.645 mM) PE for 10 h, with a volumetric productivity of 32.0 mg/l/h, specific productivity of 64.0 mg/g-enzyme/h, and molar yield of 62.1% (Fig. 5A). At this time, 0.18 mg/ml deGA-PD and 0.16 mg/ml deAX-PD as the precursors of deGAX-PD remained, whereas substrate PE and intermediates PD3, PD, deG-PD, and deA-PD disappeared. After 10 h, the concentration of deGAX-PD was not observed to increase. The enzyme also produced 0.34 mg/ml (0.434 mM) deGAX-PGD from 1 mg/ml (0.729 mM) PD3 for 8 h, with a volumetric productivity of 42.5 mg/l/h, specific productivity of 85.0 mg/g/h, and molar yield of 59.6% (Fig. 5B). The crude enzyme from *R. oryzae* produced deA-PD and deA-PGD from PE and PGD3 in the Platycodi radix extract with specific productivities of 81.9 and 37.7 mg/g/h, and molar yields of 100 and 100%, respectively [31], and the specific productivity using reagent-grade platycoside was found to be 4.8-fold higher than that obtained using platycoside in the Platycodi radix extract for the conversion of PE into PD [25]. Therefore, the specific productivities of deGAX platycosides by the crude enzyme from *A. tubingensis* were higher than those of deA platycosides by the crude enzyme from *R. oryzae*. These results suggested that the hydrolysis of all glucose residues at C-3 and apiose-xylose at C-28 was more difficult than the hydrolysis of all residues except for the innermost glucose at C-3 and apiose at C-28.

In conclusion, the crude enzyme from *A. tubingensis* converted the PE and PGD3 into deGAX-PD and deGAX-PGD by hydrolyzing all glucose residues at C-3 and apiose and xylose residues at C-28. The hydrolytic activity was increased by cultivation using citrus pectin and corn steep solid as carbon and nitrogen sources, respectively, in the culture medium. The induced crude enzyme effectively produced deGAX-PD and deGAX-PGD from PE and PGD3, respectively. To the best of our knowledge, this is the first study to report the production of deGAX-PD and deGAX-PGD from glycosylated platycosides.

## Figures and Tables

**Table 1:** Effects of carbon and nitrogen sources on the induction of deGAX platycoside-producing activity of the crude enzyme from *A. tubingensis*.

Carbon source	Relative activity (%)	Nitrogen source	Relative activity (%)
Control (CMC)	100 ± 2.1	Negative control	100 ± 1.7
Arabic gum	ND^a^	Ammonium nitrate	ND
Cellulose	66 ± 3.6	Ammonium sulfate	19 ± 0.1
Citrus pectin	133 ± 1.6	Corn steep solid	138 ± 1.6
Rice bran	52 ± 2.0	Peptone	124 ± 1.2
Sugar beet sludge	ND	Urea	ND
Wheat bran	21 ± 4.1	Yeast extract	31 ± 2.3

^a^ND: not detected.
